# Global systematic review of cost of illness and economic evaluation studies associated with snakebite

**DOI:** 10.7189/jogh.10.020415

**Published:** 2020-12

**Authors:** Chanthawat Patikorn, Doungporn Leelavanich, Ahmad Khaldun Ismail, Iekhsan Othman, Suthira Taychakhoonavudh, Nathorn Chaiyakunapruk

**Affiliations:** 1 Department of Social and Administrative Pharmacy, Faculty of Pharmaceutical Sciences, Chulalongkorn University, Bangkok, Thailand; 2 Department of Emergency Medicine, Chancellor Tuanku Muhriz Hospital, Universiti Kebangsaan Malaysia Medical Centre, Kuala Lumpur, Malaysia; 3 Neuropharmacology Research Laboratory, Jeffrey Cheah School of Medicine and Health Sciences, Monash University Malaysia, Bandar Sunway, Selangor, Malaysia; 4 College of Pharmacy, University of Utah, Salt Lake City, Utah, USA; 5 School of Pharmacy, Monash University Malaysia, Selangor, Malaysia

## Abstract

**Background:**

Snakebite envenoming, a high priority Neglected Tropical Disease categorized by the World Health Organization (WHO), has been considered as a poverty-related disease that requires greater global awareness and collaboration to establish strategies that effectively decrease economic burdens. This prompts the need for a comprehensive review of the global literature that summarizes the global economic burden and a description of methodology details and their variation. This study aimed to systematically identify studies on cost of illness and economic evaluation associated with snakebites, summarize study findings, and evaluate their methods to provide recommendations for future studies.

**Methods:**

We searched PubMed, EMBASE, Cochrane library, and Econlit for articles published from inception to 31 July 2019. Original articles reporting costs or full economic evaluation related with snakebites were included. The methods and reporting quality were assessed. Costs were presented in US dollars (US$) in 2018.

**Results:**

Twenty-three cost of illness studies and three economic evaluation studies related to snakebites were included. Majority of studies (18/23, 78.26%) were conducted in Low- and Middle-income countries. Most cost of illness studies (82.61%) were done using hospital-based data of snakebite patients. While, four studies (17.39%) estimated costs of snakebites in communities. Five studies (21.74%) used societal perspective estimating both direct and indirect costs. Only one study (4.35%) undertook incidence-based approach to estimate lifetime costs. Only three studies (13.04%) estimated annual national economic burdens of snakebite which varied drastically from US$126 319 in Burkina Faso to US$13 802 550 in Sri Lanka. Quality of the cost of illness studies were varied and substantially under-reported. All three economic evaluation studies were cost-effectiveness analysis using decision tree model. Two of them assessed cost-effectiveness of having full access to antivenom and reported cost-effective findings.

**Conclusions:**

Economic burdens of snakebite were underestimated and not extensively studied. To accurately capture the economic burdens of snakebites at both the global and local level, hospital data should be collected along with community survey and economic burdens of snakebites should be estimated both in short-term and long-term period to incorporate the lifetime costs and productivity loss due to premature death, disability, and consequences of snakebites.

Snakebite envenoming is one of the most overlooked public health issues globally. Even though almost 4.5-5.4 million people are bitten by snakes annually, snake antivenoms are still not readily and sufficiently available especially in the developing region of the world like Sub-Saharan Africa, South-East Asia and South Asia [1]. Snakebite envenoming can result in fatalities; permanent physical disabilities, such as amputation, blindness and kidney failure; and psychological symptoms, such as Post-Traumatic Stress Disorder (PTSD). In 2017, World Health Organization (WHO) has recognized the importance of snakebite envenoming and categorized it as a high priority Neglected Tropical Disease with the goal of facilitating a cooperation and collaboration across countries to establish strategies to effectively decrease the burden of snakebite envenoming [2].

To systematically establish the effective strategies to deal with snakebites as well as prioritize resources for making antivenom available, it is important to know the true burden of the public-health threat posed by snakebites. However, only a few studies have estimated the economic burdens of snakebites and include only some regions of the world [2-5]. This study aimed to summarize the global economic burden of snakebites by systematically identify studies on cost of illness and economic evaluation associated with snakebites as well as evaluate the methods used in these studies. Our findings will generate overall findings and methodological recommendations for future economic studies related to snakebites.

## METHODS

This review followed the Methodological Expectations of Cochrane Intervention Reviews (MECIR) [6] and was reported according to the Preferred Reporting Items for Systematic Reviews and Meta-Analyses (PRISMA) statement [7]. The PRISMA checklist table of this review is provided in Appendix S1 in the **Online Supplementary Document****.** The study protocol was submitted to PROSPERO for registration (CRD42020147299).

### Data source, search strategy, and eligibility criteria

We searched the following four electronic bibliographic databases; PubMed, Embase, Cochrane Library, and EconLit to identify articles related to cost of illness and economic evaluations associated with snakebites from any country which were published from inception to 31 July 2019. The search term used was snake* AND (burden OR economic* OR cost* OR “cost of illness” OR resource OR expenditure OR “economic evaluation” OR “cost-effectiveness” OR “cost-utility” OR “cost-benefit”). There was no language restriction in this review. Additional searches were done on the health economic databases including Health Economic Evaluation Database (HEED), Cost-effectiveness Analysis Registry, and Health Technology Assessment Database. The detail search strategies are provided in Appendix S2 in the Online Supplementary Document. To be included, study must meet the following inclusion criteria; original articles reporting costs associated with snakebites estimated by primary data collection and original articles of the full economic evaluations associated with snakebites.

### Study selection and data extraction

Two reviewers (CP and DL) independently performed the screening of titles and abstracts for relevance. The full-text articles of the potentially eligible studies were retrieved and selected based on the eligibility criteria by two independent reviewers (CP and DL). Data extraction were performed by two independent reviewers (CP and DL) using the data extraction form in MS Excel (Microsoft Inc, Seattle WA, USA). Discrepancies were discussed among reviewers and resolved by the third reviewer (ST). Methodological characteristics and study findings from the cost of illness studies and economic evaluations were extracted. We extracted the following data from cost of illness studies; study design, country, setting, study period/duration, sample size, perspective, data source, cost estimation method, cost components, currency year, snake species, antivenoms, and cost estimates. The following data were extracted from economic evaluation studies; target population, study perspective, comparators, time horizon, discount rate, choice of health outcomes, resource and cost estimation method, currency year, choice of model, sensitivity analyses, snake species, antivenoms, study parameters, incremental costs and outcomes.

### Quality assessment

Two independent reviewers (CP and DL) assessed the quality of the studies. Cost of illness studies were assessed using the cost-of-illness evaluation checklist by Larg and Moss [8]. Economic evaluations were assessed using the ten-item Drummond checklist [9] and the 24-item Consolidated Health Economic Evaluation Reporting Standards (CHEERS) checklist [10].

### Data synthesis

Methodological characteristics, study findings, and quality of the studies were summarized and presented. Countries were classified by income level according to the World Bank [11]. Costs were presented according to the recommendations of Turner et al., 2019 [12]. For studies that did not provide the year of cost data, the year of publication was used. Adjustment for inflation was done using the Gross Domestic Product (GDP) deflator of the studied country. Cost estimates were then converted and reported in 2018 US dollars (US$). To further facilitate comparison of costs across countries, the total costs associated with snakebites were estimated as percentage of the country’s GDP in 2018. GDP deflator, exchange rate, and GDP were obtained from the World Bank [13-15].

## RESULTS

### Study selection

**Figure 1:**
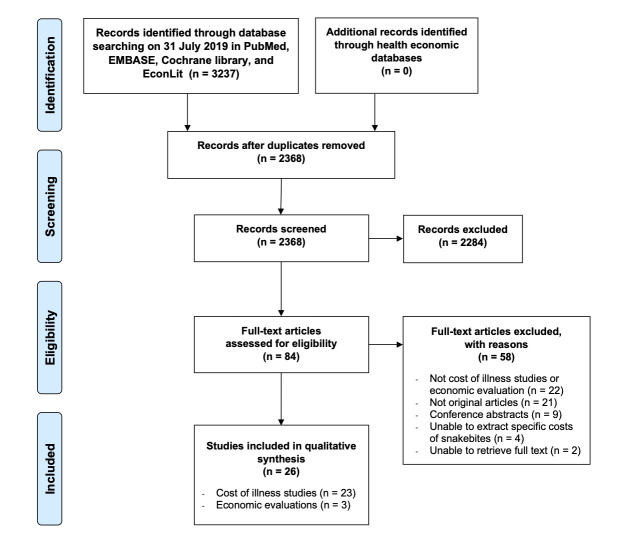
Study selection flow.

We identified 3237 articles through electronic database searches. The searches in health economic databases found no additional articles. The detailed process of electronic database searching is presented in Appendix S2 in the **Online Supplementary Document****.** We included 26 studies which met the eligibility criteria as shown in **Figure 1**. The included studies comprised of 23 cost of illness studies and 3 economic evaluations. Cost of illness studies were done in 16 countries, of which mainly comprised 13 low- and middle-income countries. Only five studies (21.74%) were conducted in high-income countries.[16-20] Economic evaluation studies were done in India, Nigeria, and 16 West African countries [21-23].

### Study characteristics

The description of the study characteristics of the included studies are presented in detail in Appendix S3-S4 in the **Online Supplementary Document**. Of the 23 cost of illness studies, only 3 studies (13.04%) estimated annual national economic burdens of snakebite (**Table 1**) [5,28,40]. Nineteen studies (82.61%) were hospital-based study as they included only snakebite patients presented at hospitals [16-20,25-29,31-34,36-40]. While the remaining four studies (17.39%) considered snakebite victims in the communities to also include those who did not reach treatment facilities eg, deaths or those who seek traditional healers [5,24,30,35]. Among these studies, only one study (4.35%) holistically collected both hospital-based and community-based data [5].

**Table 1:** Characteristics of the included cost of illness studies associated with snakebites

Author, year	Income economies	Country	Perspective	Study setting	Sample size	Study design	Direct cost estimation method	Indirect cost estimation method	Source of resource utilization data	Source of price data
**East Asia and Pacific**										
Schioldann, 2018 [24]	Lower-middle	Myanmar	Patient	Three villages in Mandalay	158 participants	Cross-sectional	Participatory appraisal methods with the communities	Not collected	Interview	Interview
**Europe and Central Asia**										
Saz-Parkinson, 2012 [20]	High	Spain	Health system	Nationwide	1649 patients	Retrospective	Analysis of Spanish hospital discharge and registry database	Not collected	Database	Listed price
**Latin America and Caribbean**										
Bachan, 2017 [25]	Upper-middle	Guyana	Societal	Five hinder land regions	57 patients	Retrospective	Analysis of Medical evacuation (medevac) database	Not collected	Database	Listed price, Literature
Sotelo, 2008 [26]	Upper-middle	Mexico	Provider	One children hospital in Northwestern Mexico	79 patients	Retrospective	Review of clinical files	Not collected	Chart	Listed price
**Middle East and North Africa**										
Nikfar, 2011 [27]	Upper-middle	Iran	Health system	Nationwide	Not reported	Retrospective	Extraction from Iranian drug affair's, drug selection committee, pharmaceuticals statistics. Direct interview with stockholders and key opinion leaders	Not collected	Database, Interview, Literature	Listed price
Mashhadi, 2017 [28]	Upper-middle	Iran	Societal	Three hospitals in Ahvaz	655 patients	Cross-sectional	Review of patients’ medical records and self-reports of specialists. Face-to-face or telephone interviews with the patients.	Review of patients’ medical records and self-reports of specialists. Face-to-face or telephone interviews with the patients. Productivity loss due to hospitalization and disability were multiplied with average wage. Productivity loss due to premature mortality was calculated using GDP per capita.	Chart, Interview	Interview, Listed price
**North America**										
Curran-Sills, 2018 [16]	High	Canada	Provider	Nationwide	99 patients	Retrospective	Review of the Health Canada Special Access Program records	Not collected	Chart	Market price
Lopoo, 1998 [18]	High	United States	Provider	One referral children hospital in Oklahoma	37 patients	Retrospective	Review of medical records	Not collected	Chart	Listed price
Narra, 2014 [19]	High	United States	Societal	Thirty-three tertiary children's hospitals	2755 patients	Retrospective	Analysis of Pediatric Health Information System database	Not collected	Database	Listed price
Fowler, 2017 [17]	High	United States	Provider	One regional hospital in Texas	146 patients	Retrospective	Review of medical records	Not collected	Database	Market price
**South Asia**										
Kasturiratne, 2017 [5]	Upper-middle	Sri Lanka	Societal	All households in nine provinces	695 victims (44 136 households)	Cross-sectional, Modelling	Representative nation-wide community-based household survey for patients’ costs. Health system costs were obtained from hospital cost accounting systems and estimates of antivenom usage	Income lost in victims and their families were derived from the representative nation-wide community-based household survey	Database, Interview	Interview, Listed price
Hasan, 2012 [29]	Lower-middle	Bangladesh	Patient	Four rural tertiary level hospitals	83 patients	Prospective	Interview using structured questionnaires	Loss of wage was derived from interview using structured questionnaires	Interview	Market price, Interview,
Vaiyapuri, 2013 [30]	Lower-middle	India	Patient	Thirty villages in rural Tami Nadu	1115 victims (7578 households)	Cross-sectional	Interview using structured questionnaires	Income loss and economic loss were derived from interview using structured questionnaires	Interview	Interview
Gupt, 2015 [31]	Lower-middle	India	Provider	One hospital in Himachal Pradesh	497 patients	Retrospective	Review of medical records	Not collected	Chart	Listed price
Meena, 2016 [32]	Lower-middle	India	Health system	One tertiary hospital in Southern Rajasthan	200 patients	Prospective	Review of medical records and patients’ interview	Not collected	Chart, Interview	Listed price
Ramanath, 2016 [33]	Lower-middle	India	Provider	One rural hospital	190 patients	Prospective, Retrospective	Review of medical records and patients’ interview	Not collected	Chart, Interview	Listed price
Qureshi, 2013 [34]	Lower-middle	Pakistan	Health system	Two public-sector hospitals	74 patients	Prospective	Review of medical records	Not collected	Chart	Listed price
Sharma, 2004 [35]	Low	Nepal	Patient	Community-based; Five villages in eastern Terai	143 victims (1817 households)	Cross-sectional	Community-based survey with interview using a standardized questionnaire	Community-based survey with interview using a standardized questionnaire	Interview	Interview
**Sub-Saharan Africa**										
Darryl, 2016 [36]	Upper-middle	South Africa	Health system	Fifty-six public hospitals in KwaZulu Natal	56 hospitals	Modelling, Retrospective	Analysis of KwaZulu Natal Department of Health central pharmacy antivenom supply data and review of hospital admission records	Not collected	Chart, Database	Listed price, Literature
Michael, 2011 [37]	Lower-middle	Nigeria	Societal	One 22-bed rural hospital in central Nigeria	72 patients	Prospective	Review of medical records and patients’ interview	Not collected	Chart, Interview	Listed price
Kasilo, 1993 [38]	Lower-middle	Zimbabwe	Provider	Six urban major referral hospitals	995 patients	Retrospective	Review of hospital records	Not collected	Chart	Listed price
Tagwireyi, 2001 [39]	Lower-middle	Zimbabwe	Provider	One large teaching hospital	78 patients	Retrospective	Review of medical records	Not collected	Chart	Market price
Gampini, 2016 [40]	Low	Burkina Faso	Patient	All public health facilities	114 126 patients	Retrospective	Number of cases were extracted from Statistical Yearbook of the Ministry of Health. Antivenom consumption data were collected from the drug wholesalers established in Burkina Faso.	Not collected	Database	Market price

Most studies (95.65%) undertook prevalence-based approach which costs of illness of all prevalent cases in the specific period of the study, usually one episode of snakebite, were estimated [5,16-20,24-27,29-40]. Only one study (4.35%) undertook incidence-based approach to estimate lifetime costs of illness including costs of productivity loss due to snakebite, disability, and premature death [28].

In terms of study perspectives, five studies (21.74%) utilized societal perspective which included both direct and indirect costs [5,19,25,28,37]. Components of indirect costs reported in the included studies were costs of productivity loss due to premature death and disability, income loss, and family income loss. Conversely, direct medical costs especially antivenom costs were estimated in all studies. Direct medical cost components estimated varied across studies. For example, traditional healer costs were reported in three studies (13.04%), [5,24,35] while six studies (26.09%) estimated direct non-medical costs including costs of transportation, communication, food, accommodation, and caregivers [5,25,27,29,33,35]. All of the reported cost components are summarized in Appendix S5 in the **Online Supplementary Document****.**

Multiple sources of information were used to quantify, and value health care resources utilized by snakebite patients. Sources of health care resource utilization data were chart, database, interview, and literature. Chart (n = 12, 52.17%) [16,18,26,28,31-34,36-39] and interview (n = 10, 43.48%) [5,24,27-30,32,33,35,37] were the most commonly used sources. Prices of health care resources were from interview, listed price, literature, and market price. Listed price was the most common source of price data (n = 15, 65.22%) [5,18-20,25-28,31-34,36-38].

Only three economic evaluation studies were identified. All of them were cost-effectiveness analysis using decision analytic models [21-23]. Two studies compared no access to antivenom to full access in envenomed snakebite patients presented to hospital [21,22]. While, another study compared antivenom alone with the antivenom adjunct combination strategy to improve the proportion of snake victims reaching health care facilities [23]. The health outcomes of snakebite in the models were similar including full recovery, death, and amputation. Lifelong was selected as the time horizon to capture deaths and disabilities. Discount was applied only to outcomes because direct costs of snakebite normally occurred during treatment in health care facilities [21-23].

### Quality assessment

Reporting quality of the included studies was assessed and presented in Appendix S6-S7 in the **Online Supplementary Document**. Reporting quality of the included cost of illness studies were substantially varied. Perspective, epidemiologic approach, health care resource valuation, and detail cost components were not clearly specified and reported. None of the included studies performed sensitivity analysis or estimated intangible costs. In contrast, reporting quality of the included economic evaluation studies was high where most aspects were met by all three studies [21-23].

### Annual national cost estimates of snakebite

Among the included cost of illness studies, three studies estimated costs of snakebites as annual national costs in Iran, Sri Lanka, and Burkina Faso [5,28,40]. **Table 2** shows the annual national cost estimates of snakebite in US$2018, cost breakdowns, and their contribution to the total costs. The number of snakebite patients ranged from 5379 patient in Iran [28] to 80 277 patients in Sri Lanka [5]. These numbers were either retrieved from annual report or extrapolated and estimated from studies. The total annual national costs of snakebite drastically varied from US$126 319 in Burkina Faso [40] to US$13 802 550 in Sri Lanka [5]. These three studies estimated the annual national economic burdens of snakebite, of which direct medical costs contributed the most to the total costs (68.01%-77.14%) followed by indirect costs (13.16%-24.86%), and direct non-medical costs (7.13%-9.70%) [5,28,40]. Moreover, the total annual national costs from three countries were then calculated as percentage of the country’s GDP in 2018 which resulted in less than 0.001% in Iran and Burkina Faso and 0.016% in Sri Lanka. Average cost estimates per patient per episode of snakebite were summarized in US$2018 in Appendix S8 in the **Online Supplementary Document**.

**Table 2:** Annual national cost estimates of snakebite in US$, 2018

Author, year	Country	Perspective	Study approach	Annual number of snakebite patients	Source of annual incident cases	Annual national cost estimates in US$2018 with cost contribution to total costs			
**Direct medical costs (%)**	**Direct non-medical costs (%)**	**Indirect costs (%)**	**Total costs**
Mashhadi, 2017 [28]	Iran	Societal	Incidence-based	5,379	Annual report	2 658 464 (68.01%)	278 665 (7.13%)	971 612 (24.86%)	3,908,741
Kasturiratne, 2017 [5]	Sri Lanka	Societal	Prevalence-based	80,277	Extrapolated from community survey and previous studies	10 647 355 (77.14%)	1 338 614 (9.70%)	181,6581 (13.16%)	13,802,550
Gampini, 2016 [40]	Burkina Faso	Patients	Prevalence-based	22,337	Estimated from previous studies	126 319 (100.00%)	NR	NR	126,319

N/A – not applicable, NR – not reported

### Findings of economic evaluation studies associated with snakebite

Two studies reported outcomes as Disability-adjusted life years (DALYs) and deaths from snakebite [21,22], while the other study reported only DALYs [23]. All three studies concluded that their interventions were very-cost-effective because the Incremental Cost-Effectiveness Ratio (ICER) per DALY averted of these studies ranged from 69.87 to 256.62 US$, which were much below the willingness-to-pay threshold of one GDP per capita of US$351.60 to US$2504.14 in the study countries [21-23]. While, the ICER per death averted of two studies ranged from US$1634.40 to US$5666.75 [21,22]. Costs of antivenom [21,22] and proportion of patients with severe envenomation [23] were the most sensitive parameters (Appendix S9 in the **Online Supplementary Document**).

## DISCUSSION

Accurate and comprehensive estimations of economic burdens of snakebites are highly needed to demonstrate the real impact of this neglected tropical disease. Revealing the economic burdens of snakebites will make the policymakers understand the magnitude and contribution of each cost component. Moreover, the cost estimates derived can be further utilized in the subsequent economic evaluation studies which accurate cost estimates will result in less uncertain economic models. Thus, strategies and resources could be better developed and allocated to effectively deal with snakebites.

This review is the first systematic review which comprehensively identified economic studies related to snakebites in published literature. The methodological characteristics and study findings were summarized. Our review found that 23 cost of illness studies and 3 economic evaluations had been conducted so far. Majority of these studies were conducted in Low- and Middle-income countries in regions highly inhabited by snakes. However, the overall methods of the included cost of illness studies related to snakebites were not comprehensive as most of them estimated only non-national direct costs in the hospital setting from non-societal perspectives.

Based on our review findings, several methodological issues should be considered for future research on economic burden estimation. First, the economic burden studies of snakebites should be done from the societal perspective in the national level to fully capture both direct and indirect costs and their relevant cost components. Our review found that collecting only direct medical costs could only capture 68.01%-77.14% of the national annual total costs of snakebites. Direct non-medical costs and indirect costs contribute 7.13%-9.07% and 13.16%-24.86%, respectively [5,28,40].

Second, economic burden studies should capture all snakebite victims by using both hospital-based and community-based data to ensure that those not seeking medical care are included. Hospital-based studies mostly captured envenomed or severe snakebite victims who were more likely to go to hospital. Therefore, incorporating the community-based survey could further improve the completeness of the economic burdens because not all of the victims could reach hospital. They may die beforehand due to long travel distances, be referred to higher level health care facilities, or seek traditional healers for help due to cultural belief [5,24,30,35]. For example, it was found that approximately 45.2% of snakebite victims in Sri Lanka consulted traditional healers which could further delay access to effective antivenom and result in worse outcomes [41]. Therefore, victim transportation and treatment seeking behavior should also be incorporated into the analysis depending on each country. If national epidemiological data of snakebites is lacking, data collection could be done in a representative group of snakebite victims then appropriately extrapolate to national cost estimates.

Third, although snakebites are episodic and most costs occur during the first few weeks, economic burdens of snakebites should be estimated both in short-term and long-term period to take into account the lifetime costs and productivity loss due to premature death and disability. Estimating indirect costs only in the short-term period as income loss might underestimate the indirect costs of snakebites. The contribution of indirect cost estimates to the total costs increased from 13.16% to 24.86% when long-term costs of productivity loss due to premature death and disability from snakebite were incorporated [5,28,40].

Lastly, consequences of snakebites should be broader to include all relevant disabilities and their following costs and productivity loss such as premature death, amputation, blindness, kidney failure, malignant ulcers, pregnancy loss, scarring, and PTSD [21]. These will be varied by species of venomous snakes within each country. Therefore, all important snake species and their geographical distribution should also be considered to capture all relevant costs and consequences of snakebites.

Our systematic review has several limitations that should be discussed. The quality assessment of the included cost of illness studies could only be done in the aspects of reporting quality, since there are no guidelines or checklist to directly evaluate the methodological quality of the cost of illness studies. Nonetheless, articles with good reporting quality could imply their methodological quality to some extent. Moreover, the global economic burdens of snakebites and country comparison could not be estimated due to the underestimated nature of snakebite economic burdens revealed from our review. Further research should be conducted using both hospital-based and community-based data to gather and highlight the overlooked global economic burdens of this neglected tropical disease taking into account our methodological recommendations.

## CONCLUSION

Economic burdens of snakebite were underestimated and not extensively studied. Majority of studies only provided direct costs of snakebite patients presented to the hospitals. There was a lack of study estimating national economic burdens of snakebites. Due to likely underestimated economic burden, hospital data should be used to combine with community survey to ensure the accurate estimation of overall economic burdens of snakebite victims. Having full access to antivenom was found to be very cost-effective. Future studies should focus on how to make antivenoms available and affordable to snakebite victims.

## Additional material

Online Supplementary Document
