# Supplementary material for: Global systematic review of cost of illness and economic evaluation studies associated with snakebite
Source: J Glob Health. 2020 Nov 6;10(2):020415. doi: 10.7189/jogh.10.020415 (PMC7719278; doi:10.7189/jogh.10.020415)
Supplement: Online Supplementary Document [file jogh-10-020415-s001.pdf]

SUPPLEMENTARY MATERIAL

GLOBAL SYSTEMATIC REVIEW OF COST OF ILLNESS AND ECONOMIC EVALUATION STUDIES ASSOCIATED WITH SNAKEBITE

Appendix S1 *PRISMA checklist.*

Section/topic	#	Checklist item	Reported on page #
**TITLE**			
Title	1	Identify the report as a systematic review, meta-analysis, or both.	**1**
**ABSTRACT**			
Structured summary	2	Provide a structured summary including, as applicable: background; objectives; data sources; study eligibility criteria, participants, and interventions; study appraisal and synthesis methods; results; limitations; conclusions and implications of key findings; systematic review registration number.	**1-2**
**INTRODUCTION**			
Rationale	3	Describe the rationale for the review in the context of what is already known.	**3**
Objectives	4	Provide an explicit statement of questions being addressed with reference to participants, interventions, comparisons, outcomes, and study design (PICOS).	**3**
**METHODS**			
Protocol and registration	5	Indicate if a review protocol exists, if and where it can be accessed (e.g., Web address), and, if available, provide registration information including registration number.	**3**
Eligibility criteria	6	Specify study characteristics (e.g., PICOS, length of follow-up) and report characteristics (e.g., years considered, language, publication status) used as criteria for eligibility, giving rationale.	**3**
Information sources	7	Describe all information sources (e.g., databases with dates of coverage, contact with study authors to identify additional studies) in the search and date last searched.	**3**
Search	8	Present full electronic search strategy for at least one database, including any limits used, such that it could be repeated.	**3-4, Appendix S2**

Section/topic	#	Checklist item	Reported on page #
Study selection	9	State the process for selecting studies (i.e., screening, eligibility, included in systematic review, and, if applicable, included in the meta-analysis).	**3-4**
Data collection process	10	Describe method of data extraction from reports (e.g., piloted forms, independently, in duplicate) and any processes for obtaining and confirming data from investigators.	**3-4**
Data items	11	List and define all variables for which data were sought (e.g., PICOS, funding sources) and any assumptions and simplifications made.	**3-4**
Risk of bias in individual studies	12	Describe methods used for assessing risk of bias of individual studies (including specification of whether this was done at the study or outcome level), and how this information is to be used in any data synthesis.	**4**
Summary measures	13	State the principal summary measures (e.g., risk ratio, difference in means).	**N/A**
Synthesis of results	14	Describe the methods of handling data and combining results of studies, if done, including measures of consistency (e.g., I^2) for each meta-analysis.	**N/A**
Risk of bias across studies	15	Specify any assessment of risk of bias that may affect the cumulative evidence (e.g., publication bias, selective reporting within studies).	**-**
Additional analyses	16	Describe methods of additional analyses (e.g., sensitivity or subgroup analyses, meta-regression), if done, indicating which were pre-specified.	**N/A**
**RESULTS**			
Study selection	17	Give numbers of studies screened, assessed for eligibility, and included in the review, with reasons for exclusions at each stage, ideally with a flow diagram.	**4, Figure 1**
Study characteristics	18	For each study, present characteristics for which data were extracted (e.g., study size, PICOS, follow-up period) and provide the citations.	**4-5, Table 1, Appendix S3-S4**
Risk of bias within studies	19	Present data on risk of bias of each study and, if available, any outcome level assessment (see item 12).	**Appendix S6-S7**
Results of individual studies	20	For all outcomes considered (benefits or harms), present, for each study: (a) simple summary data for each intervention group (b) effect estimates and confidence intervals, ideally with a forest plot.	**Appendix S5**
Synthesis of results	21	Present results of each meta-analysis done, including confidence intervals and measures of consistency.	**N/A**

Section/topic	#	Checklist item	Reported on page #
Risk of bias across studies	22	Present results of any assessment of risk of bias across studies (see Item 15).	**-**
Additional analysis	23	Give results of additional analyses, if done (e.g., sensitivity or subgroup analyses, meta-regression [see Item 16]).	**N/A**
**DISCUSSION**			
Summary of evidence	24	Summarize the main findings including the strength of evidence for each main outcome; consider their relevance to key groups (e.g., healthcare providers, users, and policy makers).	**7-8**
Limitations	25	Discuss limitations at study and outcome level (e.g., risk of bias), and at review-level (e.g., incomplete retrieval of identified research, reporting bias).	**8**
Conclusions	26	Provide a general interpretation of the results in the context of other evidence, and implications for future research.	**8**
**FUNDING**			
Funding	27	Describe sources of funding for the systematic review and other support (e.g., supply of data); role of funders for the systematic review.	**8**

From: Moher D, Liberati A, Tetzlaff J, Altman DG, The PRISMA Group (2009). Preferred Reporting Items for Systematic Reviews and Meta-Analyses: The PRISMA Statement. PLoS Med 6(7): e1000097. doi:10.1371/journal.pmed1000097

Appendix S2 *Full search strategies.*

Searches were conducted for articles published up to 31 July 2019.

1. **PubMed**

Search number	Search terms	Results
#1	snake*	25363
#2	burden OR economic* OR cost* OR “cost of illness” OR resource OR expenditure	1481775
#3	"economic evaluation" OR "cost-effectiveness" OR "cost-utility" OR "cost-benefit"	113425
#4	#2 OR #3	1481775
#5	#1 AND #4	1317

2. **EMBASE (via Elsevier)**

Search number	Search terms	Results
#1	snake*	23162
#2	burden OR economic* OR cost* OR “cost of illness” OR resource OR expenditure	1527675
#3	"economic evaluation" OR "cost-effectiveness" OR "cost-utility" OR "cost-benefit"	214963
#4	#2 OR #3	1527675
#5	#1 AND #4	1783

3. **Cochrane library**

Search number	Search terms	Results
#1	snake*	458
#2	burden OR economic* OR cost* OR “cost of illness” OR resource OR expenditure	108006
#3	"economic evaluation" OR "cost-effectiveness" OR "cost-utility" OR "cost-benefit"	26054
#4	#2 OR #3	108006
#5	#1 AND #4	48

4. **EconLit (via EBSCO)**

Search number	Search terms	Results
#1	snake*	107
#2	burden OR economic* OR cost* OR “cost of illness” OR resource OR expenditure	1259448
#3	"economic evaluation" OR "cost-effectiveness" OR "cost-utility" OR "cost-benefit"	12663
#4	#2 OR #3	1259448
#5	#1 AND #4	89

Additional searches in health economic databases

1. **Health Economic Evaluation Database (HEED)**
   HEED ceased to publish and was inaccessible since 2014.
2. **Tuft’s Cost-effectiveness Analysis (CEA) Registry**
   Search found no result.
3. **Health Technology Assessment Database**
   Search found no result.

Appendix S3 *Methodological characteristics of the included cost of illness studies associated with snakebites.*

Region/ Income economies	Country	Author, year	Perspective	Study population	Study setting	Study period	Sample size	Study design	Study approach	Currency (Costing year)	Direct cost estimation method	Indirect cost estimation method	Source of resource utilization	Source of price
**East Asia and Pacific**														
Lower-middle	Myanmar	Schioldann, 2018 [1]	Patient	Snakebite victims	Three villages in Mandalay	2016	158 participants	CS	PB	MMK, USD (NR)	Bottom-up		Interview	Interview
**Europe and Central Asia**														
High	Spain	Saz-Parkinson, 2012 [2]	Health system	Envenomed snakebite patients	Nationwide	1997-2009	1649 patients	R	PB	EUR (NR)	Bottom-up		Database	Listed price
**Latin America and Caribbean**														
Upper-middle	Guyana	Bachan, 2017 [3]	Societal	Snakebite patients required medical evacuation	Five hinder land regions	2011-2015	57 patients	R	PB	USD (NR)	Bottom-up		Database	Listed price, Literature
Upper-middle	Mexico	Sotelo, 2008 [4]	Provider	Snakebite children	One children hospital in Northwestern Mexico	1977-2006	79 patients	R	PB	MXN (NR)	Bottom-up		Chart	Listed price
**Middle East and North Africa**														
Upper-middle	Iran	Nikfar, 2011 [5]	Health system	Patients required antidotes	Nationwide	2004-2008	N/A (national data)	R	PB	IRR (NR)	Bottom-up		Database, Interview, Literature	Listed price
Upper-middle	Iran	Mashhadi, 2017 [6]	Societal	Snakebite and scorpion sting patients	Three hospitals in Ahvaz	January to December 2015	655 patients	CS	IB	I$ (NR)	Bottom-up	Human-capital	Chart, Interview	Interview, Listed price
**North America**														
High	Canada	Curran-Sills, 2018 [7]	Provider	Snakebite patients	Nationwide	January 2008 to April 2016	99 patients	R	PB	USD (2017)	Bottom-up		Chart	Market price
High	United States	Lopoo, 1998 [8]	Provider	Snakebite children	One referral children hospital in Oklahoma	1987-1997	37 patients	R	PB	USD (NR)	Bottom-up		Chart	Listed price
High	United States	Narra, 2014 [9]	Societal	Envenomed children	Thirty-three tertiary children's hospitals	2009	2755 patients	R	PB	USD (NR)	Bottom-up		Database	Listed price
High	United States	Fowler, 2017 [10]	Provider	Crotaline Snakebite patients	One regional hospital in Texas	January 2010 to November 2014	146 patients	R	PB	USD (NR)	Bottom-up		Database	Market price

Region/ Income economies	Country	Author, year	Perspective	Study population	Study setting	Study period	Sample size	Study design	Study approach	Currency (Costing year)	Direct cost estimation method	Indirect cost estimation method	Source of resource utilization	Source of price
**South Asia**														
Upper-middle	Sri Lanka	Kasturiratne, 2017 [11]	Societal	Snakebite victims	All households in nine provinces	August 2012 to June 2013	695 victims (44,136 households)	CS, M	PB	LKR, USD (NR)	Bottom-up	Human-capital	Database, Interview	Interview, Listed price
Lower-middle	Bangladesh	Hasan, 2012 [12]	Patient	Snakebite patients	Four rural tertiary level hospitals	June to October 2006	83 patients	P	PB	USD (NR)	Bottom-up	Human-capital	Interview	Market price, Interview,
Lower-middle	India	Vaiyapuri, 2013 [13]	Patient	Snakebite victims	Thirty villages in rural Tami Nadu	Novembe r to Decembe r 2010	1115 victims (7578 households)	CS	PB	EUR, INR (NR)	Bottom-up	Human-capital	Interview	Interview
Lower-middle	India	Gupt, 2015 [14]	Provider	Snakebite patients	One hospital in Himachal Pradesh	January 2008 to Decembe r 2012	497 patients	R	PB	INR, USD (NR)	Bottom-up		Chart	Listed price
Lower-middle	India	Meena, 2016 [15]	Health system	All patients	One tertiary hospital in Southern Rajasthan	2014-2015	200 patients	P	PB	INR (NR)	Bottom-up		Chart, Interview	Listed price
Lower-middle	India	Ramanath, 2016 [16]	Provider	Snakebite patients	One rural hospital	January 2011 to February 2015	190 patients	P, R	PB	INR (NR)	Bottom-up		Chart, Interview	Listed price
Lower-middle	Pakistan	Qureshi, 2013 [17]	Health system	Envenomed snakebite adults	Two public-sector hospitals	June to Septemb er 2010	74 patients	P	PB	PKR (NR)	Bottom-up		Chart	Listed price
Low	Nepal	Sharma, 2004 [18]	Patient	Snakebite victims	Community-based; Five villages in eastern Terai	Decembe r 2001	143 victims (1817 households)	CS	PB	USD (NR)	Bottom-up	Human-capital	Interview	Interview
**Sub-Saharan Africa**														
Upper-middle	South Africa	Darryl, 2016 [19]	Health system	Snakebite patients	Fifty-six public hospitals in KwaZulu Natal	2012-2013	56 hospitals	M, R	PB	USD (NR)	Bottom-up		Chart, Database	Listed price, Literature
Lower-middle	Nigeria	Michael, 2011 [20]	Societal	Snakebite patients	One 22-bed rural hospital in central Nigeria	April to July 2006	72 patients	P	PB	NGN, USD (NR)	Bottom-up		Chart, Interview	Listed price
Lower-middle	Zimbabwe	Kasilo, 1993 [21]	Provider	Snakebite patients	Six urban major referral hospitals	1980-1989	995 patients	R	PB	USD, ZWD (NR)	Bottom-up		Chart	Listed price

Region/ Income economies	Country	Author, year	Perspective	Study population	Study setting	Study period	Sample size	Study design	Study approach	Currency (Costing year)	Direct cost estimation method	Indirect cost estimation method	Source of resource utilization	Source of price
Lower-middle	Zimbabwe	Tagwireyi, 2001 [22]	Provider	Uncomplicated envenomed snakebite patients	One large teaching hospital	January 1996 to December 1999	78 patients	R	PB	USD, ZWD (2000)	Bottom-up		Chart	Market price
Low	Burkina Faso	Gampini, 2016 [23]	Patient	Snakebite patients	All public health facilities	2010-2014	N/A (national data)	R	PB	USD (NR)	Bottom-up		Database	Market price

CS – Cross-sectional, EUR – Euro, I$ - International Dollar, IB – Incidence-based, INR – Indian Rupee, IRR – Iranian Rial, LKR – Sri Lankan Rupee, M – Modelling, MMK – Myanmar Kyat, MXN – Mexican Peso, N/A – Not Applicable, NGN – Nigerian Naira, NR – Not reported, PB – Prevalence-based, PKR – Pakistani Rupee, R – Retrospective, USD – United States Dollar, ZWD – Zimbabwean Dollar

Description of methodological characteristics of included cost of illness studies

Perspective

Eight studies undertook analysis from the healthcare provider's perspective [4,7,8,10,14,16,21,22], and five studies utilized health system's perspective. [2,5,15,17,19] All of these studies only focused on direct medical costs such as antivenom costs, and hospitalization costs. Other five studies utilized patient's perspective [1,12,13,18,23] and the remaining five studies utilized societal perspective. [3,6,9,11,20] These studies incorporated broader scope of costs including both direct and indirect costs.

Study population and setting

Most studies included population from all age groups. Only three studies focused on children [4,8,9], and one studies focused on adult.[17] Studies were categorized from study location as hospital-based and community-based study. Nineteen studies were hospital-based study as they focused only snakebite patients presented at hospitals.[2-10,12,14-17,19-23] While the remaining four studies focused on snakebite victims in the communities to also include those who did not reach treatment facilities.[1,11,13,18]

Study design and study approach

Study design was classified as cross-sectional, modelling, prospective, and retrospective. Twelve studies collected data retrospectively using data in medical records or databases. [2-5,7-10,21-23] Cross-sectional studies were done in four studies.[1,6,13,18] Four studies prospectively collected data from snakebite patients. [12,15,17,20] One study collected data both retrospectively and prospectively.[16] The remaining two studies collected data which were further used in modelling costs of snakebite.[11,19]

Study approach for cost of illness study includes prevalence-based and incidence-based approach. Prevalent-based approach estimates cost of illness of all prevalent cases in the specific period of the study, usually one episode of snakebite. While, incidence-based estimates lifetime costs including costs related to disability or sequelae due to the diseases. Most studies undertook prevalence-based approach [1-5,7-23], except one study which estimated costs of productivity loss due to disease and to premature death using incidence-based approach.[6]

Costing year and reported currency

Year of cost estimation was reported only in two studies.[7,22] The costing years of the other 21 studies were not reported. Therefore, they were imputed using the publication year. [1-6,8-21,23] Cost estimates were reported in local currencies in nine studies [2,4,8-10,15-17], international currencies in six studies [3,6,7,12,18,19,23], and International Dollars (a hypothetical currency unit that is designed to capture differences in relative prices across different settings) in one study. [6] The other seven studies reported their results in both local and international currencies. [1,11,13,14,20-22]

Direct cost estimation method

Direct costs are commonly quantified using top-down or bottom-up approach. The top-down approach estimates direct costs by allocating aggregate costs at the national level according to the resources used by the disease cases. The bottom-up approach quantifies direct costs by calculating the resources used by the disease cases at the patient level. The costs per patient quantified using both approaches can be then extrapolated to national costs using relevant epidemiological data.[24] In this review, all of the included studies utilized bottom-up approach. [1-23]

Indirect cost estimation method

Indirect costs are costs of productivity losses due to premature death or consequences of the disease. Indirect costs are normally estimated by either human-capital or friction cost approach. Human-capital approach values the productivity losses as output lost due inability to fully perform productive activities. While, the friction cost approach values the productivity losses as the employment costs to replace the ill workers.[24] Only five of the included cost of illness studies estimated indirect costs due to snakebite, all of which utilized human-capital approach.[6,11-13,18] Therefore, only these studies could be considered as economic burden study because they estimated both direct and indirect costs

Sources of healthcare resource utilization

Sources of healthcare resource utilization are defined as Chart, Database, Interview, and Literature. Chart includes patient medical records. Database includes electronic medical records, claim databases, and national registries. Interview is conducted using questionnaire. Literature includes articles in published or unpublished sources and government documents. Studies might use more than one source of information. Chart was the most used source of healthcare resource utilization (n=12) [4,6-8,14-17,19-22], followed by interview (n=10) [1,5,6,11-13,15,16,18,20], database (n=7) [2,3,5,9,10,19,23], and literature (n=1).[5]

Sources of price of healthcare resource

Sources of price of healthcare resource are defined as Interview, Listed price, Literature, and Market price. Interview includes prices or costs of illness revealed by interview especially out of pocket costs paid by patients and families. Listed price includes prices set in the hospital and financial or accounting databases. Literature includes standard cost database and published articles. Market price includes retail and wholesale prices. Studies might use more than one source of information. Listed price was assumed to be used when studies utilized hospital records to quantify resource utilization but not clearly stated the price source. Listed price was the most used source of price of healthcare resource (n=15) [2-6,8,9,11,14-17,19-21], followed by interview (n=6) [1,6,11-13,18], market price (n=5) [7,10,12,22,23], and literature (n=2).[3,19]

Appendix S4 *Methodological characteristics of the included economic evaluation studies associated with snakebites.*

Author, year	Country	Study Setting	Target population	Type of snake	Type of antivenom	Intervention	Comparator	Type of EE	Type of model	Study perspective	Health outcomes	Time horizon	Discount rate	Sensitivity analysis
Habib, 2015 [25]	Nigeria	Public healthcare facilities in Nigeria	Envenomed snakebite victims	- Carpet viper<br>- non-carpet viper snakes	- Monovalent<br>- Polyvalent	Availability of geographically appropriate and effective antivenoms	No availability of effective antivenoms	CEA	Decision analytic model	Public healthcare system	- Full recovery<br>- Amputation<br>- Death	Lifelong	3% only outcomes	- One-way<br>- Best-worst<br>- Scenario
Hamza, 2016 [26]	16 West Africa countries	Public healthcare facilities in West Africa	Envenomed snakebite victims	- Vipers<br>- non-viper snakes	- Monovalent<br>- Polyvalent	Availability of effective antivenoms	No availability of effective antivenoms	CEA	Decision analytic model	Public healthcare system	- Full recovery<br>- Amputation<br>- Death	Lifelong	3% only outcomes	- One-way<br>- PSA<br>- Scenario
Herzel, 2018 [27]	India	Prehospital setting and Private health-care providers in South India	Snakebite victims	Any snakes	Not reported	Antivenom/adjunct combination strategy with supportive care	Antivenom and supportive care	CEA	Decision analytic model	Private healthcare provider	- Full recovery<br>- Finger/Toe amputation<br>- Below-knee amputation<br>- Death	Lifelong	3% only outcomes	- One-way<br>- PSA<br>- Scenario

CEA – Cost-effectiveness analysis, PSA – Probabilistic sensitivity analysis

Description of methodological characteristics of economic evaluation studies

All three included economic evaluation studies are cost-effectiveness analysis utilized decision analytic models.[25-27] Two studies compared no access to antivenom to full access from the public healthcare system's perspective. These studies only focused on envenomed snakebite patients presented to hospital. [25,26] While, another study compared antivenom alone with the antivenom adjunct combination strategy to improve the proportion of victims reaching healthcare facilities from the private healthcare system's perspective. This study focused on snakebite victims outside of the hospital.[27] Antivenoms were part of the analysis in all three studies. The health outcomes of snakebite in the models were similar including full recovery, death, and amputation. Lifelong was selected as the time horizon to capture deaths and disabilities. However, discount was applied only to outcomes because direct costs of snakebite normally occurred during treatment in healthcare facilities. All three studies performed sensitivity analyses.[25-27]

Appendix S5 *Cost components reported in the included cost of illness studies associated with snakebites.*

Region/ Income economies	Country	Author, year	Direct medical costs											Direct non-medical costs					Indirect costs			
			Antivenom cost	Antibiotic cost	Other medicine cost	Hospitalization cost	Diagnosis cost	Health worker and service cost	Laboratory cost	Medical product cost	Traditional healer cost	Forfeited antivenom cost	Medical evacuation cost	Transportation cost	Communication cost	Food cost	Accommodation cost	Caregiver cost	Cost of productivity loss	Income lost in employed victim	Income loss in self-employed victims	Family income loss
**East Asia and Pacific**																						
Lower-middle	Myanmar	Schioldann, 2018 [1]	+	-	-	-	-	-	-	-	+	-	-	-	-	-	-	-	-	-	-	-
**Latin America and the Caribbean**																						
High	Spain	Saz-Parkinson, 2012 [2]	+	-	-	+	-	-	-	-	-	-	-	-	-	-	-	-	-	-	-	-
**Middle East and North Africa**																						
Upper-middle	Guyana	Bachan, 2017 [3]	+	-	+	-	-	-	-	+	-	-	+	+	-	+	+	-	-	-	-	-
Upper-middle	Mexico	Sotelo, 2008 [4]	+	+	-	+	-	-	-	-	-	-	-	-	-	-	-	-	-	-	-	-
Upper-middle	Iran	Nikfar, 2011 [5]	+	+	+	+	+	+	+	+	-	-	-	+	+	+	+	-	+	-	-	-
Upper-middle	Iran	Mashhadi, 2017 [6]	+	-	-	-	-	-	-	-	-	-	-	-	-	-	-	-	-	-	-	-
**North America**																						
High	Canada	Curran-Sills, 2018 [7]	+	-	-	-	-	-	-	-	-	+	-	-	-	-	-	-	-	-	-	-
High	United States	Lopoo, 1998 [8]	+	-	-	-	-	-	-	-	-	-	-	-	-	-	-	-	-	-	-	-
High	United States	Narra, 2014 [9]	+	-	-	+	-	-	-	-	-	-	-	-	-	-	-	-	-	-	-	-
High	United States	Fowler, 2017 [10]	+	+	-	+	+	-	+	-	-	-	-	-	-	-	-	-	-	-	-	-
**South Asia**																						
Upper-middle	Sri Lanka	Kasturiratne, 2017 [11]	+	-	+	+	-	-	+	+	+	-	-	+	+	+	-	+	-	+	+	+
Lower-middle	Bangladesh	Hasan, 2012 [12]	+	+	+	-	-	+	-	-	-	-	-	-	+	+	+	+	-	+	-	-
Lower-middle	India	Vaiyapuri, 2013 [13]	+	-	-	-	-	-	-	-	-	-	-	-	-	-	-	-	-	-	-	+
Lower-middle	India	Gupt, 2015 [14]	+	-	-	-	-	-	-	-	-	-	-	-	-	-	-	-	-	-	-	-
Lower-middle	India	Meena, 2016 [15]	+	+	+	-	-	-	-	-	-	-	-	-	-	-	-	-	-	-	-	-

Region/ Income economies	Country	Author, year	Direct medical costs											Direct non-medical costs					Indirect costs			
			Antivenom cost	Antibiotic cost	Other medicine cost	Hospitalization cost	Diagnosis cost	Health worker and service cost	Laboratory cost	Medical product cost	Traditional healer cost	Forfeited antivenom cost	Medical evacuation cost	Transportation cost	Communication cost	Food cost	Accommodation cost	Caregiver cost	Cost of productivity loss	Income lost in employed victim	Income loss in self-employed victims	Family income loss
Lower-middle	India	Ramanath, 2016 [16]	+	+	+	-	-	-	-	-	-	-	-	+	-	-	-	-	-	-	-	-
Lower-middle	Pakistan	Qureshi, 2013 [17]	+	-	-	-	-	-	-	-	-	-	-	-	-	-	-	-	-	-	-	-
Low	Nepal	Sharma, 2004 [18]	+	+	+	+	-	+	-	-	+	-	-	+	-	-	-	-	-	+	-	-
**Sub-Saharan Africa**																						
Upper-middle	South Africa	Darryl, 2016 [19]	+	-	-	+	-	-	-	-	-	-	-	-	-	-	-	-	-	-	-	-
Lower-middle	Nigeria	Michael, 2011 [20]	+	-	-	+	-	-	-	-	-	-	-	-	-	-	-	-	-	-	-	-
Lower-middle	Zimbabwe	Kasilo, 1993 [21]	+	-	-	+	-	-	-	-	-	-	-	-	-	-	-	-	-	-	-	-
Lower-middle	Zimbabwe	Tagwireyi, 2001 [22]	+	+	-	-	-	-	-	-	-	-	-	-	-	-	-	-	-	-	-	-
Low	Burkina Faso	Gampini, 2016 [23]	+	-	-	-	-	-	-	-	-	-	-	-	-	-	-	-	-	-	-	-
**No. of studies reporting cost component**			**23**	**8**	**7**	**10**	**2**	**3**	**3**	**3**	**3**	**1**	**1**	**5**	**3**	**4**	**3**	**2**	**1**	**3**	**1**	**2**
**Percentage (%)**			**100**	**34.78**	**30.43**	**43.48**	**8.70**	**13.04**	**13.04**	**13.04**	**13.04**	**4.35**	**4.35**	**21.74**	**13.04**	**17.39**	**13.04**	**8.70**	**4.35**	**13.04**	**4.35**	**8.70**

Description of reported cost components of snakebite

Direct medical costs

Direct medical costs were estimated in all studies. Medicine costs for the treatment of snakebite were commonly reported. Anti-snake venom costs were estimated in all studies, antibiotic costs in eight studies (34.78%) [4,6,9,12,13,16,18,22], and other medicine costs such as analgesics in seven studies (30.43%).[3,6,11-13,16,18] Hospitalization costs were also reported in ten studies (43.48%).[2,4,6,8,9,11,18-21] The other cost components were varied by studies. For example, traditional healer costs were reported in three studies (13.04%). [1,11,18]

Direct non-medical costs

Six studies estimated direct non-medical costs [3,5,11,12,16,18]. Components of direct non-medical cost reported in the included studies were costs of transportation, communication, food, accommodation, and caregivers. Transportation and food costs were the most commonly reported components among the six studies.

Indirect costs

Five studies estimated indirect costs. [6,11-13,18] Components of indirect costs reported in the included studies were costs of productivity loss due to premature death and disability, income loss, and family income loss. Income loss in employed snakebite victims were the most commonly reported components among the five studies.

Appendix S6 *Quality assessment of the included cost of illness studies associated with snakebites.*

**Author, year**	**Specified perspective**	**Specified Epidemiologic approach**	**Specified study question**	**Specified Resource quantification method**	**Specified Healthcare resource valuation**	**Specified Productivity loss valuation**	**Estimated Intangible cost**	**Description of statistical analyses**	**Performed sensitivity analysis**	**Reporting of detailed cost components**
Kasilo, 1993 [21]	NO	NO	NO	YES	NO	NO	NO	YES	NO	NO
Lopoo, 1998 [8]	NO	NO	NO	YES	NO	NO	NO	YES	NO	NO
Tagwireyi, 2001 [22]	NO	NO	YES	YES	YES	NO	NO	YES	NO	YES
Sharma, 2004 [18]	NO	NO	YES	YES	YES	YES	NO	YES	NO	YES
Sotelo, 2007 [4]	NO	NO	YES	YES	YES	NO	NO	YES	NO	NO
Michael, 2011 [20]	NO	NO	YES	YES	NO	NO	NO	YES	NO	NO
Nikfar, 2011 [5]	NO	NO	NO	YES	YES	NO	NO	YES	NO	YES
Hasan, 2012 [12]	NO	NO	YES	YES	YES	YES	NO	YES	NO	YES
Saz-Parkinson, 2012 [2]	NO	NO	YES	YES	NO	NO	NO	YES	NO	NO
Qureshi, 2012 [17]	NO	NO	YES	YES	NO	NO	NO	NO	NO	NO
Vaiyapuri, 2013 [13]	NO	NO	YES	YES	YES	NO	NO	YES	NO	NO
Narra, 2014 [9]	NO	NO	YES	YES	YES	NO	NO	YES	NO	NO
Gupt, 2015 [14]	NO	NO	NO	YES	NO	NO	NO	YES	NO	NO
Darryl, 2016 [19]	NO	NO	YES	YES	YES	NO	NO	YES	NO	YES
Gampini, 2016 [23]	NO	NO	YES	YES	YES	NO	NO	YES	NO	NO
Meena, 2016 [15]	NO	NO	NO	YES	NO	NO	NO	YES	NO	NO
Ramanath, 2016 [16]	NO	NO	NO	YES	YES	NO	NO	YES	NO	NO
Bachan, 2017 [3]	NO	NO	YES	YES	NO	NO	NO	NO	NO	YES

Author, year	Specified perspective	Specified Epidemiologic approach	Specified study question	Specified Resource quantification method	Specified Healthcare resource valuation	Specified Productivity loss valuation	Estimated Intangible cost	Description of statistical analyses	Performed sensitivity analysis	Reporting of detailed cost components
Fowler, 2017 [10]	NO	NO	YES	YES	YES	NO	NO	YES	NO	NO
Kasturiratne, 2017 [11]	NO	NO	YES	YES	YES	YES	NO	YES	NO	YES
Mashhadi, 2017 [6]	YES	YES	YES	YES	YES	YES	NO	YES	NO	YES
Curran-Sills, 2018 [7]	NO	NO	YES	YES	YES	NO	NO	YES	NO	NO
Schioldann, 2018 [1]	NO	NO	NO	NO	NO	NO	NO	NO	NO	NO
Kasilo, 1993 [21]	NO	NO	NO	YES	NO	NO	NO	YES	NO	NO
**No. of fulfilling studies**	**1**	**1**	**16**	**22**	**14**	**4**	**0**	**20**	**0**	**8**
**Percentage**	**4.35%**	**4.35%**	**69.57%**	**95.65%**	**60.87%**	**17.39%**	**0.00%**	**86.96%**	**0.00%**	**34.78%**

Appendix S7 *Quality assessment of included economic evaluation studies associated with snakebites*.

**Recommended aspects**	**Author, year**			**No. of fulfilling studies**	**Percentage (%)**
	**Habib, 2015 [25]**	**Hazam, 2016 [26]**	**Herzel, 2018 [27]**		
Study question	YES	YES	YES	3	100.00
Description of intervention and comparator	YES	YES	NO	2	66.67
Measurement of effectiveness	YES	YES	NO	2	66.67
Assumption of costs and outcomes	YES	YES	YES	3	100.00
Currency and price data	YES	NO	YES	2	66.67
Choice of model	YES	YES	YES	3	100.00
Perspective	YES	YES	YES	3	100.00
Time horizon	YES	YES	YES	3	100.00
Discount rate	YES	YES	YES	3	100.00
Calculated and reported ICER	YES	YES	YES	3	100.00
Sensitivity analysis	YES	YES	YES	3	100.00
Disclosed funding source	YES	YES	YES	3	100.00

Appendix S8 *Cost estimates per episode of snakebite in US$ 2018.*

Region/ Income economies	Country	Author, year	Perspective	Average cost per episode of snakebite (US$ 2018)			
				Direct medical costs	Direct non-medical costs	Indirect costs	Total costs
**East Asia and Pacific**							
Lower-middle	Myanmar	Schioldann, 2018 [1]	Patient	230.80		NR	230.80
**Latin America and the Caribbean**							
High	Spain	Saz-Parkinson, 2012 [2]	Health system	2339.40	NR	NR	2339.40
**Middle East and North Africa**							
Upper-middle	Guyana	Bachan, 2017 [3]	Societal	1090.20	1170.91	NR	2261.11
Upper-middle	Mexico	Sotelo, 2008 [4]	Provider	962.34	NR	NR	962.34
Upper-middle	Iran	Nikfar, 2011 [5]	Health system	NR	NR	NR	NR
Upper-middle	Iran	Mashhadi, 2017 [6]	Societal	494.23	546.04	180.63	1220.90
**North America**							
High	Canada	Curran-Sills, 2018 [7]	Provider	25553.86	NR	NR	25553.86
High	United States	Lopoo, 1998 [8]	Provider	3592.27	NR	NR	3592.27
High	United States	Narra, 2014 [9]	Societal	1296.74	NR	NR	1296.74
High	United States	Fowler, 2017 [10]	Provider	40493.10	NR	NR	40493.10
**South Asia**							
Upper-middle	Sri Lanka	Kasturiratne, 2017 [11]	Societal	123.60	19.32	26.20	169.12
Lower-middle	Bangladesh	Hasan, 2012 [12]	Societal	106.59	66.89	19.68	193.16
Lower-middle	India	Vaiyapuri, 2013 [13]	Patient	0.00 - 6034.10		34.48 - 1724.03	NR
Lower-middle	India	Gupt, 2015 [14]	Provider	80.91	NR	NR	80.91
Lower-middle	India	Meena, 2016 [15]	Health system	176.37	NR	NR	176.37
Lower-middle	India	Ramanath, 2016 [16]	Provider	522.47	NR	NR	522.47
Lower-middle	Pakistan	Qureshi, 2013 [17]	Health system	78.85	NR	NR	78.85
Low	Nepal	Sharma, 2004 [18]	Patient	68.98	11.76	41.30	122.02
**Sub-Saharan Africa**							
Upper-middle	South Africa	Darryl, 2016 [19]	Health system	1295.63	NR	NR	1295.63
Lower-middle	Nigeria	Michael, 2011 [20]	Societal	8.44	NR	NR	8.44
Lower-middle	Zimbabwe	Kasilo, 1993 [21]	Provider	4.32	NR	NR	4.32
Lower-middle	Zimbabwe	Tagwireyi, 2001 [22]	Provider	4.33	NR	NR	4.33
Low	Burkina Faso	Gampini, 2016 [23]	Patients	NR	NR	NR	NR

NR – Not reported

Appendix S9 *Summary of findings of included economic evaluation studies associated with snakebites.*

Author, year	Country	Sequalae (rate)	Currency, year	Antivenom price/dose (US$ 2018)	Antivenom price/course (US$ 2018)	Disability weight	Reported outcome	GDP per capita (US$ 2018)	Threshold used	ICER (US$ 2018)	Study conclusion	Most sensitive parameters
Habib, 2015 [25]	Nigeria	- Amputation (3%)<br>- Blindness (0.01%)*<br>- PTSD (20%)*	US$, 2015	US$87.68	US$87.68	- Amputation 0.102<br>- Blindness 0.552*<br>- PTSD 0.105*	DALYs, Deaths	US$1090.70	1 GDP per capita	- US$1634.40 per Death averted<br>- US$69.87 per DALY averted	Very cost-effective	- Costs of antivenom<br>- Proportion of envenoming due to carpet viper,<br>- Probability of dying following carpet viper envenoming
Hamza, 2016 [26]	16 West Africa countries	- Amputation (3%)<br>- Blindness (0.01%)*<br>- PTSD (20%)*	US$, 2015	US$139.73	US$139.73	- Amputation 0.102<br>- Blindness 0.552*<br>- PTSD 0.105*	DALYs, Deaths	US$351.60 to US$2504.14	1 GDP per capita	- US$1823.77 to US$5666.75 per Death averted<br>- US$75.80 to US$256.62 per DALY averted	Very cost-effective	- Costs of antivenom<br>- Antivenom effectiveness against non-carpet viper envenoming<br>- Probability of dying following non-carpet viper envenoming
Herzel, 2018 [27]	India	- Amputation (25%)	US$, 2015	US$10.01	US$300.44	- Below-knee amputation 0.164<br>- Finger/toe amputation 0.02	DALYs	US$1569.72	1 GDP per capita	- US$71.16 per DALYs averted	Very cost-effective	- Proportion of severe envenomation<br>- Cost of severe envenomation<br>- Proportion of patients reaching treatment facilities

* - included in model in sensitivity analysis, DALYs – Disability-Adjusted Life Years, GDP – Gross Domestic Product, ICER – Incremental Cost-Effectiveness Ratio, PTSD – Post-Traumatic Stress Disorder

REFERENCES

1 Schioldann E, Mahmood MA, Kyaw MM, Halliday D, Thwin KT, Chit NN, et al. Why snakebite patients in Myanmar seek traditional healers despite availability of biomedical care at hospitals? Community perspectives on reasons. PLoS neglected tropical diseases. 2018;12:e0006299.

2 Saz-Parkinson Z, del Pino Luengo M, Lopez-Cuadrado T, Andujar D, Carmona-Alferez R, Flores RM, et al. Approach to the epidemiology of venomous bites in Spain. Toxicon : official journal of the International Society on Toxinology. 2012;60:706-11.

3 Bachan V, Tatkan G, Adu-Krow W. Transportation (medical evacuations) of snake bitten persons from the Hinterland Regions in Guyana: Cost analysis. West Indian Medical Journal. 2017;66:43.

4 Sotelo N. Review of treatment and complications in 79 children with rattlesnake bite. Clinical pediatrics. 2008;47:483-9.

5 Nikfar S, Khatibi M, Abdollahi-Asl A, Abdollahi M. Cost and utilization study of antidotes: An Iranian experience. International Journal of Pharmacology. 2011;7:46-9.

6 Mashhadi I, Kavousi Z, Peymani P, Salman Zadeh Ramhormozi S, Keshavarz K. Economic burden of scorpion sting and snake bite from a social perspective in Iran. Shiraz E Medical Journal. 2017;18.

7 Curran-Sills G, Kroeker J. Venomous Snakebites in Canada: A National Review of Patient Epidemiology and Antivenom Usage. Wilderness & environmental medicine. 2018;29:437-45.

8 Lopoo JB, Bealer JF, Mantor PC, Tuggle DW. Treating the snakebitten child in North America: a study of pit viper bites. Journal of pediatric surgery. 1998;33:1593-5.

9 Narra A, Lie E, Hall M, Macy M, Alpern E, Shah SS, et al. Resource utilization of pediatric patients exposed to venom. Hospital pediatrics. 2014;4:276-82.

10 Fowler AL, Hughes DW, Muir MT, VanWert EM, Gamboa CD, Myers JG. Resource Utilization After Snakebite Severity Score Implementation into Treatment Algorithm of Crotaline Bite. The Journal of emergency medicine. 2017;53:854-61.

11 Kasturiratne A, Pathmeswaran A, Wickremasinghe AR, Jayamanne SF, Dawson A, Isbister GK, et al. The socio-economic burden of snakebite in Sri Lanka. PLoS neglected tropical diseases. 2017;11:e0005647.

12 Hasan SM, Basher A, Molla AA, Sultana NK, Faiz MA. The impact of snake bite on household economy in Bangladesh. Tropical doctor. 2012;42:41-3.

13 Vaiyapuri S, Vaiyapuri R, Ashokan R, Ramasamy K, Nattamaisundar K, Jeyaraj A, et al. Snakebite and its socio-economic impact on the rural population of Tamil Nadu, India. PloS one. 2013;8:e80090.

14 Gupt A, Bhatnagar T, Murthy BN. Epidemiological profile and management of snakebite cases - A cross sectional study from Himachal Pradesh, India. Clinical Epidemiology and Global Health. 2015;3:S96-S100.

15 Meena VK, Atray M, Agrawal A. Evaluation of drug utilization pattern in indoor patients of medicine department at tertiary care teaching hospital in southern Rajasthan. International Journal of Pharmaceutical Sciences and Research. 2016;7:3835-40.

16 Ramanath KV, Anitha C, Junied S. Study on snake bite poisoning and it's outcomes in a Rural Tertiary Care Hospital. Research Journal of Pharmaceutical, Biological and Chemical Sciences. 2016;7:1875-89.

17 Qureshi H, Alam SE, Mustufa MA, Nomani NK, Asnani JL, Sharif M. Comparative cost and efficacy trial of Pakistani versus Indian anti snake venom. JPMA The Journal of the Pakistan Medical Association. 2013;63:1129-32.

18 Sharma SK, Chappuis F, Jha N, Bovier PA, Loutan L, Koirala S. Impact of snake bites and determinants of fatal outcomes in Southeastern Nepal. American Journal of Tropical Medicine and Hygiene. 2004;71:234-8.

19 Darryl W, Sartorius B, Hift R. Estimating the Burden of Snakebite on Public Hospitals in KwaZulu Natal, South Africa. Wilderness & environmental medicine. 2016;27:53-61.
20 Michael GC, Thacher TD, Shehu MI. The effect of pre-hospital care for venomous snake bite on outcome in Nigeria. Transactions of the Royal Society of Tropical Medicine and Hygiene. 2011;105:95-101.
21 Kasilo OM, Nhachi CF. A retrospective study of poisoning due to snake venom in Zimbabwe. Human & experimental toxicology. 1993;12:15-8.
22 Tagwireyi DD, Ball DE, Nhachi CF. Routine prophylactic antibiotic use in the management of snakebite. BMC clinical pharmacology. 2001;1:4.
23 Gampini S, Nassouri S, Chippaux JP, Semde R. Retrospective study on the incidence of envenomation and accessibility to antivenom in Burkina Faso. The journal of venomous animals and toxins including tropical diseases. 2016;22:10.
24 Larg A, Moss JR. Cost-of-illness studies: a guide to critical evaluation. PharmacoEconomics. 2011;29:653-71.
25 Habib AG, Lamorde M, Dalhat MM, Habib ZG, Kuznik A. Cost-effectiveness of antivenoms for snakebite envenoming in Nigeria. PLoS neglected tropical diseases. 2015;9:e3381.
26 Hamza M, Idris MA, Maiyaki MB, Lamorde M, Chippaux JP, Warrell DA, et al. Cost-Effectiveness of Antivenoms for Snakebite Envenoming in 16 Countries in West Africa. PLoS neglected tropical diseases. 2016;10:e0004568.
27 Herzel BJ, Samuel SP, Bulfone TC, Raj CS, Lewin M, Kahn JG. Snakebite: An Exploratory Cost-Effectiveness Analysis of Adjunct Treatment Strategies. The American journal of tropical medicine and hygiene. 2018;99:404-12.
